# LRP5 Is Required for Vascular Development in Deeper Layers of the Retina

**DOI:** 10.1371/journal.pone.0011676

**Published:** 2010-07-20

**Authors:** Chun-hong Xia, Zipora Yablonka-Reuveni, Xiaohua Gong

**Affiliations:** 1 School of Optometry and Vision Science Program, University of California, Berkeley, California, United States of America; 2 Department of Biological Structure, University of Washington School of Medicine, Seattle, Washington, United States of America; Cincinnati Children's Hospital Medical Center, United States of America

## Abstract

**Background:**

The low-density lipoprotein receptor-related protein 5 (LRP5) plays an important role in the development of retinal vasculature. LRP5 loss-of-function mutations cause incomplete development of retinal vessel network in humans as well as in mice. To understand the underlying mechanism for how LRP5 mutations lead to retinal vascular abnormalities, we have determined the retinal cell types that express LRP5 and investigated specific molecular and cellular functions that may be regulated by LRP5 signaling in the retina.

**Methods and Findings:**

We characterized the development of retinal vasculature in LRP5 mutant mice using specific retinal cell makers and a GFP transgene expressed in retinal endothelial cells. Our data revealed that retinal vascular endothelial cells predominantly formed cell clusters in the inner-plexiform layer of LRP5 mutant retina rather than sprouting out or migrating into deeper layers to form normal vascular network in the retina. The IRES-β-galactosidase (LacZ) report gene under the control of the endogenous LRP5 promoter was highly expressed in Müller cells and was also weakly detected in endothelial cells of the retinal surface vasculature. Moreover, the LRP5 mutant mice had a reduction of a Müller cell-specific glutamine transporter, Slc38a5, and showed a decrease in b-wave amplitude of electroretinogram.

**Conclusions:**

LRP5 is not only essential for vascular endothelial cells to sprout, migrate and/or anastomose in the deeper plexus during retinal vasculature development but is also important for the functions of Müller cells and retinal interneurons. Müller cells may utilize LRP5-mediated signaling pathway to regulate vascular development in deeper layers and to maintain the function of retinal interneurons.

## Introduction

Retinal vasculature is essential for the transport of oxygen and nutrients to the inner retina and the subsequent removal of waste products. Mouse retinal vasculature develops through a vascular plexus from the optic nerve around birth. The primary surface vessel plexus reaches the retina periphery within 8 days after birth, and the three inter-connected and parallel vessel networks in the nerve fiber layer and the inner-plexiform and outer-plexiform layers are formed within 2 weeks [1]. The deeper plexus of the retinal vasculature develops by sprouting angiogenesis from the primary plexus [2], [3] The retinal vasculature becomes fully mature in mice at 6 weeks of age.

Wnt signaling has been emerging as one of the key regulators in retinal vasculature development. Low-density lipoprotein receptor-related protein 5 (LRP5) is an essential component of the Wnt ligand-receptor complex. A typical Wnt ligand-receptor complex consists of a ligand (Wnt or Norrin), a receptor (Frizzled) and a co-receptor (LRP5 or LRP6). Loss-of-function mutations in Norrin (an unconventional Wnt ligand), Wnt receptor Frizzled 4 (FZD4) or co-receptor LRP5, can cause familial exudative vitreoretinopathy (FEVR) in humans [4], [5], [6]. FEVR is a genetically heterogeneous eye disease characterized by incomplete and abnormal retinal vascularization, which also displays ischemic areas and leaky vessels in the retina. Remarkably, FZD4 and Norrin knockout mice develop severe defects in retinal vasculature, including obvious changes of primary surface vessels [7], [8], [9]; we have found that LRP5 knockout mice display incomplete vasculature only in the deeper plexus of the retina [10]. The underlying mechanism is unclear for why LRP5 knockout mice show much milder vascular defects in comparison to FZD4 or Norrin knockout mice. It is also unknown whether or how the properties or functions of endothelial cells and other retinal cells are altered in the LRP5 mutant retina. The Sca1-GFP tansgene [11], [12], [13] allows the direct visualization of endothelial cells during the development of retinal vasculature. We have determined specific molecular and visual functional changes that are associated with Müller cells or interneurons in LRP5 mutant retinas. These results suggest a new role for LRP5 in Müller cells besides a previous assumption for its role in endothelial cells. This study supports a new hypothesis that LRP5-mediated Wnt signaling in Müller cells regulates vascular development in the deeper plexus of the retina.

## Results

### A loss of LRP5 causes endothelial cell clustering in the inner-plexiform layer

**Figure 1:**
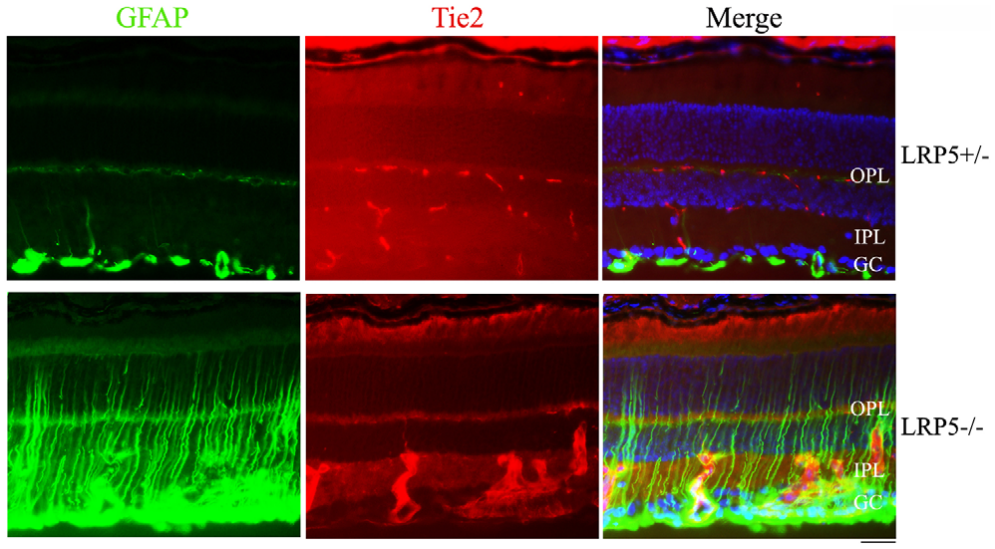
Endothelial cells form thick clusters in the LRP5 mutant retina. Co-immunostaining was performed on retinal frozen sections of 3-week-old LRP5 littermates with endothelial cell specific Tie2 (red) and glial cell specific GFAP (green) antibodies. Cell nuclei were labeled with DAPI (blue). The upper panels show normal distribution of endothelial cells and astrocytes in the control LRP5+/− retinal section. In the mutant LRP5−/− retinal section, the lower panels show endothelial cell clustering in the inner-plexiform layer and strong GFAP signal spanning the retina. OPL: outer-plexiform layer; IPL: inner-plexiform layer; GC: ganglion cell layer. Scale bar: 50 µm.

The absence of retinal vessel network in the outer plexiform layers of both LRP5 knockout (LRP5−/−) and homozygous *Lrp5^r18^* mutant retinas has been reported previously [10]. In order to investigate the underlying mechanism, we performed co-immunostaining with anti-Tie2 and anti-glial fibrillary acidic protein (GFAP) antibodies on the frozen sections of LRP5 mutant retinas. In the heterozygous (LRP5+/−) retina, endothelial cell specific Tie2 protein distribution was normal and the vascular network was observed in the outer-plexiform, inner-plexiform and ganglion cell layers; in addition, glial cell specific GFAP protein expression was restricted to astrocytes located in retinal ganglion cell layer (Figure 1, upper panels). However, in the mutant LRP5−/− retina, Tie2-positive clusters were observed in ganglion cell and inner-plexiform layers, but Tie2 signal was rarely detected in the outer-plexiform layer (Figure 1, lower panels). Moreover, upregulated GFAP protein expression was obviously detected in LRP5−/− retina (Figure 1, lower panels). These data suggest that Tie2-positive endothelial cells probably are clustered in the ganglion cell and inner-plexiform layers rather than sprouting into the outer-plexiform layer. Therefore, we investigated the development of retinal vasculature by using Sca1-GFP transgenic mice that allow the direct visualization of GFP-positive vascular endothelial cells [11], [12], [13].

**Figure 2:**
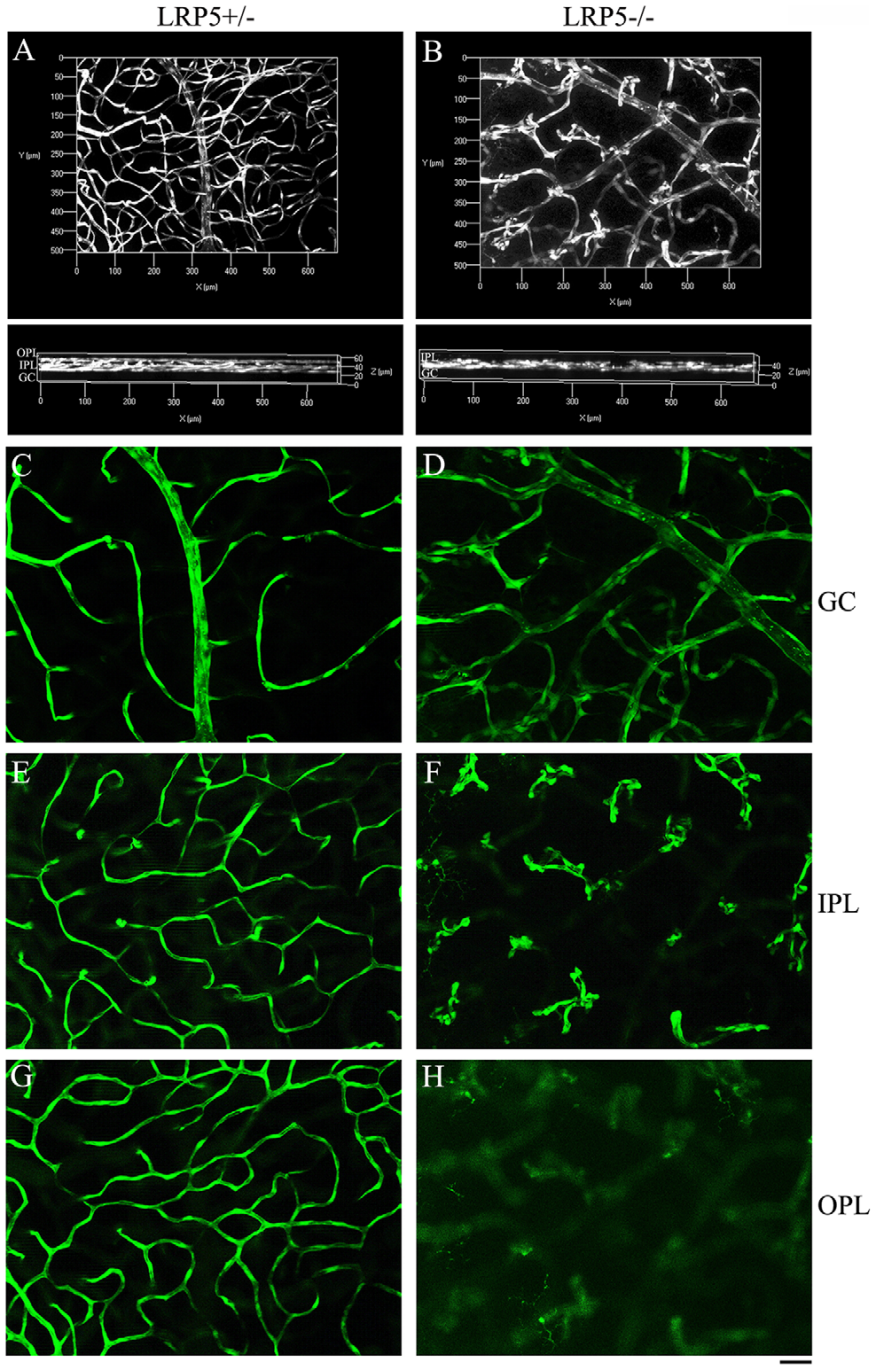
Endothelial cells are unable to form vascular network in the outer- and inner-plexiform layers of LRP5 mutant mice. A comparison of retinal vasculature visualized by Sca1-GFP positive endothelial cells in 4-week-old LRP5 littermates. 3D images of control LRP5+/− (**A**) and mutant LRP5−/− (**B**) are shown. The upper panels are front-views of 3D retinal vasculature and the lower panels are images rotated 90 degrees relative to the upper panels. Single images (**C** to **H**) corresponding to the surface vessels (labeled as GC for ganglion cell layer) and vessels in two deeper layers (IPL: inner-plexiform layer; OPL: outer plexiform layer). (**C**, **E**, **G**) images for control LRP5+/− and (**D**, **F**, **H**) for mutant LRP5−/−. Note a complete 3-layer network in the control (**A**, lower panel) but a lack of vessels in the IPL and OPL of LRP5−/− (**B**, lower panel). LRP5+/− retina shows normal capillary network in the IPL and OPL (**E** and **G**) but LRP5−/− retina displays mainly GFP-positive endothelial cell clusters in the IPL (**F**) and almost no GFP-positive endothelial cells in the OPL (**H**).

LRP5 mutant mice with the Sca1-GFP transgene were generated by mating LRP5 knockout mice and Sca1-GFP transgenic mice. Retinal vasculature was evaluated based on the three dimensional distribution of GFP-positive endothelial cells in whole-mount retinas of 4-week-old LRP5 littermates examined under a Zeiss fluorescent microscope with Apotome. GFP-positive endothelial cells of the LRP5+/− mice showed a normal retinal vasculature with many vessels branching into smaller capillary networks located in three different retinal layers, the ganglion cell nerve fiber layer (GC), the inner-plexiform layer (IPL) and the outer-plexiform layer (OPL) (Figure 2A). Moreover, GFP images taken from the LRP5+/− retina showed normal vessel network in the ganglion cell nerve fiber layer (GC), the inner-plexiform layer (IPL) and the outer-plexiform layer (OPL) (Figure 2C, 2E, 2G). However, mutant LRP5−/− retinal vasculature had thickened vessels, reduced branches and endothelial cell clusters (Figure 2B). GFP images of the LRP5−/− retina showed thicker vascular branches in the GC layer (Figure 2D), endothelial cell clusters in the IPL (Figure 2F), and a lack of the endothelial cells in the OPL (Figure 2H).

Thus, the examination of Sca1-GFP transgene expression not only confirmed the results obtained from immunostaining of endothelial cells but also allowed us to precisely observe the distribution of endothelial cells and to monitor the development of retinal vasculature. These data indicate that mutant LRP5−/− endothelial cells form clusters instead of sprouting out to form the deeper plexus of the vascular network. Thus, LRP5 signaling is important for the sprouting of endothelial cells into the deeper layers in the retina. It remains unclear whether LRP5 plays a role in endothelial cells or in surrounding cells to regulate the sprouting of endothelial cells in the retina.

### LRP5 is predominantly expressed in Müller cells

**Figure 3:**
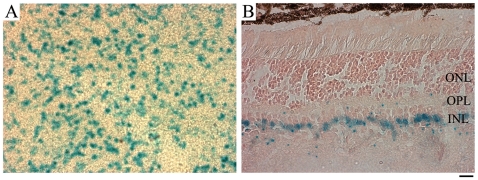
LacZ-positive cells are predominantly localized in the retinal inner nuclear layer. (**A**) The flat-mounted retina of a 3-month-old LRP5−/− mouse shows blue LacZ positive cells. (**B**) LacZ signals are observed in the cell bodies located in the inner nuclear layer of a 6-week-old LRP5−/− mouse. Cell nuclei were labeled with Fast Red. ONL: photoreceptor outer nuclear layer; OPL: outer-plexiform layer; INL: inner nuclear layer. Scale bar: 20 µm.

In order to investigate the role of LRP5 in endothelial cells or other retinal cells, we determined the cellular localization of LRP5 protein in the retina. Using several LRP5 antibodies from various vendors, we were unable to obtain conclusive results based on immunostaining signals. Thus, an indirect approach was used to study the cellular localization of LRP5. The LRP5 knockout allele has an IRES-β-galactosidase gene inserted under the control of the endogenous LRP5 promoter [14], thus the transcriptional activity of LRP5 gene can be reflected by the knockin cytosolic LacZ signal. Flat-mount retinas were stained for the LacZ signal, and positive cells were observed throughout the retinas (Figure 3A). Sagital sections of LacZ stained LRP5−/− retinas showed LacZ positive cell bodies specifically located in the inner nuclear layer (Figure 3B). We also observed weak LacZ signals in the vessels near the ganglion cell layer (data not shown), but not in the deeper layers. To determine which specific cell type expresses LRP5 in the retinal inner nuclear layer, we carried out co-immunostaining using specific cell marker antibodies in combination with the LacZ staining.

**Figure 4:**
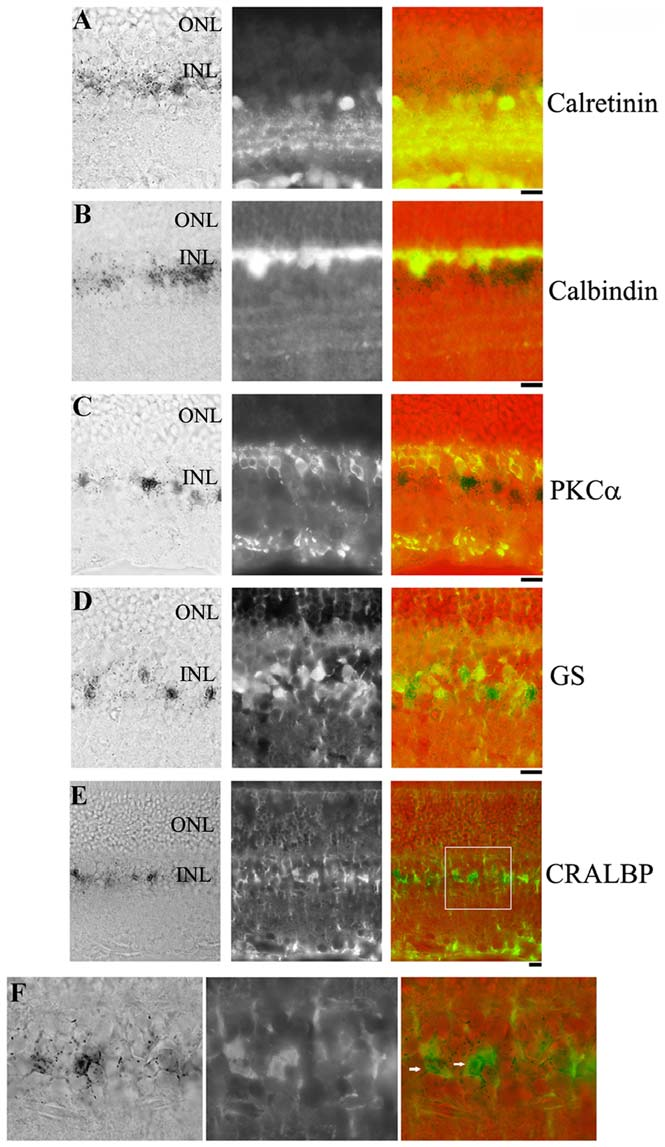
LacZ positive cells are co-localized with Müller cells but not with horizontal, amacrine and bipolar cells. Retinal frozen sections from 10-month-old LRP5+/− mice were stained for LacZ and various retinal cell specific antibodies. The left panels show LacZ-stained cells in black, the middle panels are co-immunostained images of various antibodies, and the right panels are merged images of LacZ (blue), retinal cell markers (green) and pseudo background (red). Anti-calretinin antibody staining for amacrine cells (**A**), anti-calbindin antibody staining for horizontal cells (**B**), anti-PKCα antibody staining for bipolar cells (**C**), and anti-glutamine synthetase (GS) antibody and anti-CRALBP antibody staining for Müller cells (**D** and **E**). (D) Immunostaining with retinal Müller cell specific glutamine synthetase (GS) antibody shows co-localization with LacZ signals. (E) Another Müller cell specific antibody against CRALBP also reveals its co-localization with LacZ signal. (**F**) Enlarged view of the boxed region in (E), showing cells with co-localized signals of LacZ and anti-CRALBP (indicated by white arrows). ONL: photoreceptor outer nuclear layer; INL: inner nuclear layer. Scale bars: 20 µm.

Immunostaining with cell type specific antibodies was performed to determine the specific type of cells that expresses LacZ in the LRP5+/− retina. Antibodies recognizing specific protein markers of various cells in the inner nuclear layer, including anti-PKCα for bipolar cells, anti-calretinin for amacrine cells, anti-calbindin for horizontal cells, and anti-CRALBP and anti-glutamine synthetase (GS) for Müller cells, were used. We found that anti-GS and anti-CRALBP staining for Müller specific signals revealed obvious co-localization with LacZ staining in the inner nuclear layer (Figure 4D, 4E, 4F). LacZ staining did not show any co-localization with calretinin, calbindin or PKCα (Figure 4A, 4B, 4C). These data suggest that LRP5 is highly expressed in Müller cells.

**Figure 5:**
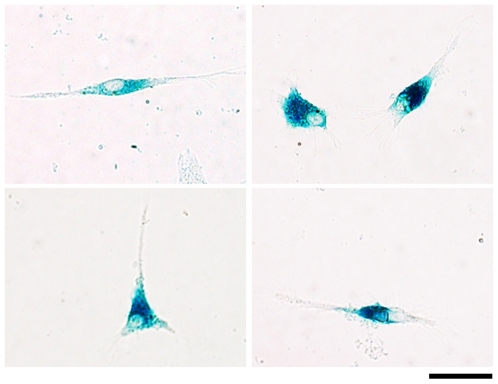
Cultured primary Müller cells from 8-day-old LRP5−/− retinas are positively stained for LacZ. Cells were cultured in coverslip for 5 days, fixed with 0.5% glutaraldehyde, and stainied for LacZ. Cytosolic LacZ signals are observed in Muller cell bodies. Scale bar, 20 µm.

To further characterize the identity of LacZ-positive cells, we isolated Müller cells from 8 days old LRP5−/− mutant retinas. After 5 days in culture, mutant primary Müller cells were stained for LacZ (Figure 5). LacZ staining was observed in the cell bodies of mostly spindle shaped cells, while the blue signal was not obvious in the cell process. The cytosolic LacZ staining in primary culture cells agrees with the LacZ signal in Müller cell bodies located in the retinal inner nuclear layer.

### Müller cell specific Slc38a5 expression is decreased in the LRP5 mutant retinas

In order to understand the underlying molecular mechanism for the abnormal vasculature observed in the LRP5 mutant retina, we examined some selective genes that are known to be important for the vasculature development and to be regulated by the Wnt signaling pathway.

**Figure 6:**
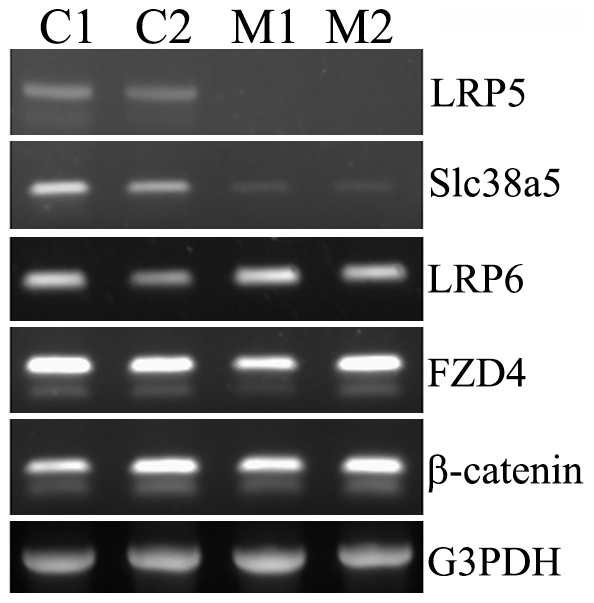
Expression of a Müller cell specific glutamine transporter, Slc38a5, is suppressed in LRP5−/− retinas. A litter of four mice at the age of 3 weeks including two control LRP5+/− (C1 and C2) and two mutant LRP5−/− (M1 and M2) was used for the experiment. As expected, no LRP5 PCR fragment was detected in the knockout retinas. The expression level of Slc38a5 was significantly reduced in the LRP5−/− retinas compared to the LRP5+/−. No consistent changes of expression level of LRP6, FZD4 and β-catenin were observed.

Semi-quantitative reverse transcription-PCR was performed to determine the mRNA expression levels of select molecules in retina. Total RNA was isolated from 3-week-old mice and cDNAs were synthesized. Among the molecules examined, we found that Müller cell specific glutamine transporter, Slc38a5 (solute carrier family 38, member 5), was significantly reduced (∼22 folds) in LRP5−/− retinas compared to wild-type or littermate heterozygous controls (Figure 6). This Slc38a5 reduction was also confirmed in homozygous *Lrp5^r18^* mutant retinas (data not shown). The expression levels of LRP6, FZD4 and β-catenin remain unchanged or show inconsistent changes (Figure 6). We were unable to observe repeatable or consistent changes of other molecules examined, including VEGF, Tie2, Flt1, Flk1, TSP1, Angiopoietin-1, Dkk3, Dll4, and Notch1 (data not shown). Thus, LRP5 is important for stimulating or maintaining the expression of Slc38a5 gene in Müller cells.

### ERG b-wave amplitude is reduced in *Lrp5^r18^* mutant mice

**Figure 7:**
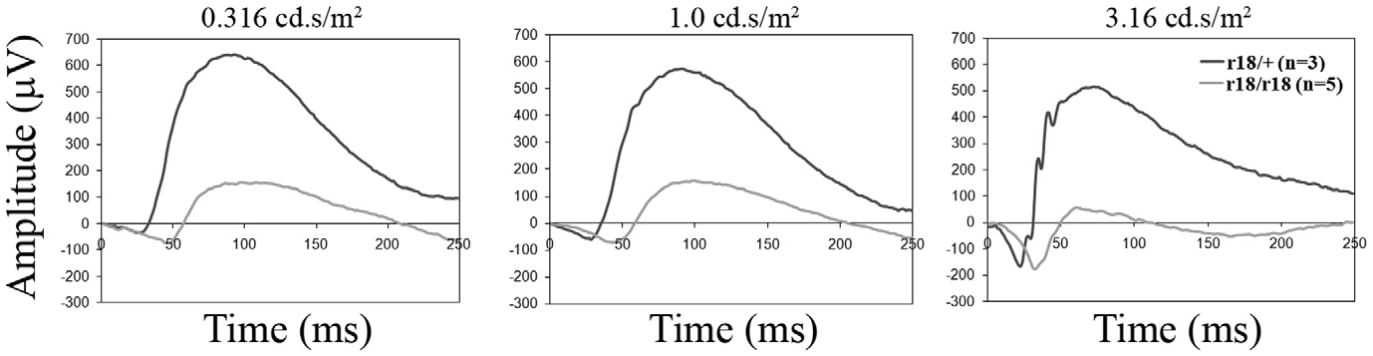
Electroretinogram (ERG) reveals reduced b-wave amplitude in the LRP5 loss-of-function mutant mice. 7-week-old LRP5^r18^ littermates including 3 heterozygous control (r18/+) and 5 homozygous mutant (r18/r18) were recorded. The results were collected by an Espion visual testing system (Diagnosys), and average amplitude value is shown for both control and mutant groups. Note a reduction of b-wave amplitude with light stimulation intensity from 0.01–3.16 cd.s/m^2^, while no obvious reduction of a-wave amplitude was observed.

In order to fully understand the consequence of various molecular and cellular changes caused by LPR5 mutations in the retina, the visual function of LRP5 mutant mice was examined by electroretinography (ERG). Compared to heterozygous *Lrp5^r18^* littermate controls, the homozygous *Lrp5^r18^* mutant mice showed normal a-wave amplitude but significantly reduced b-wave amplitude (Figure 7). Thus, a loss of LRP5 resulted in compromised functions of retinal interneurons.

## Discussion

LRP5 mutant mice are unable to form a normal vascular capillary network in the deeper plexiform layers of the retina. Both immunostaining results of vascular endothelial cell-specific Tie2 and 3D reconstruction of GFP-positive endothelial cells indicate that clustering of vascular endothelial cells, instead of normal sprouting and migrating and/or anastomosing, is the cause of incomplete retinal vasculature in the deeper layers of LRP5 mutant retina. Thus, LRP5 signaling is essential for regulating the sprouting, migrating and/or anastomosing of endothelial cells in the deeper layers of the retina. Based on the expression of knockin LacZ gene under the control of endogenous LRP5 promoter, LRP5 is predominantly expressed in retinal Müller cells although its expression is also detected in retinal endothelial cells. It is unclear whether LRP5 in Müller cells, in endothelial cells or in both cell types mediates a signal to regulate the properties of endothelial cells.

Müller cells span the entire retina and their processes are in close contact with retinal blood vessels and interneurons. Müller cells produce factors capable of modulating vascular formation, vessel permeability, the integrity of blood-retinal barrier, and the activity and survival of other retinal neurons. Studies have shown that VEGF, a stimulator for angiogenesis, is expressed transiently by Müller cells in the retinal inner nuclear layer, closely preceding the formation of the deeper plexus of retinal vasculature [15]. A recent study demonstrates that VEGF and its receptors VEGFR1 and VEGFR2 are expressed in Müller cells [16]. Müller cells have also been suggested playing a role in inducing blood-retinal barrier properties in the deeper plexus [2]. Changes in Müller cells have been detected in patients with type 1 diabetes [17]. GFAP over-expression in Müller cells is an early marker of retinal injury in retinal ischemia [18]. In the human retina during early diabetes, retinal Müller cells change from quiescent to an injury-associated phenotype and express high levels of GFAP [17]. Many angiogenic and anti-angiogenic factors are produced by Müller cells. An increased level of VEGF and a decreased level of pigment epithelium-derived factor (PEDF) have been observed in cultured retinal Müller cells treated by high glucose [19], indicating that Müller cells may contribute to unbalanced levels of VEGF and PEDF in diabetic retinopathy.

The Müller cell-specific Slc38a5 shows significantly reduced transcription level in the LRP5 mutant retinas. Slc38a5 is a sodium coupled neutral amino acid transporter mainly responsible for the glutamine uptake in retinal Müller cells [20]. Decreased expression of Slc38a5 mRNA is also reported in Norrin knockout mice [21]. Similar to LRP5, Norrin is also predominantly expressed in the Müller cells [22]. Interestingly, a previous study has shown that the loss of Müller cell specific glutamate transporter GLAST leads to reduced b-wave amplitude [23], suggesting the involvement of Müller cells in generating the b-wave of ERG. It is possible that a loss of LRP5 causes impaired Müller cells, thus leading to abnormal b-wave of ERG.

A recent study suggests that Norrin in the Müller cells activates FZD4 in endothelial cells [22]. FZD4 is known to be important for the development of retinal vasculature including the vessels in the surface ganglion cell layer. LRP5 is a co-receptor for FDZ4. Similar to the loss of FDZ4, one can assume that a functional loss of LRP5 in endothelial cells will result in a defect of retinal vasculature. However, LRP5 knockout mice display a relatively normal vascular network in the ganglion cell layer. This difference in phenotype may be because LRP5 function is either dispensable or compensated by the presence of LRP6 in endothelial cells. It remains unclear why LRP5 is essential for the development of vasculature in the retinal deeper layers where LRP6 cannot compensate its function. Moreover, it is unknown whether co-presence of LRP5 and FDZ4 in the same cell is a prerequisite for LRP5 acting as a co-receptor of FDZ4.

We hypothesize that LRP5 mediates an essential signal in Müller cells to regulate retinal vasculature in the deeper plexus. The absence of LRP5 in Müller cells disrupts this signaling pathway, thus the endothelial cells do not receive the proper signals to form the vessel network in deeper retinal layers. This hypothesis may explain why only endothelial cells in the inner- and outer-plexiform layers rely on the LRP5-mediated signaling to sprout, form the lumen, branch and anastomose. LRP6 in endothelial cells cannot compensate the loss of LRP5 in Müller cells. This hypothesis also suggests the intricate interactions among Wnts, Norrin, LRP5/6, and FZD4 between endothelial cells and Müller cells. Future research will be needed to address the underlying molecular mechanism that precisely controls the patterning of retinal vessels in different layers and to understand how Müller cells provide guiding cues to the retinal vessel network in the deep layers of retina.

## Materials and Methods

### Animals

All studies and examinations were conducted in accordance with a protocol for the Use of Animals in Research (protocol number: R280–1210), approved by the Animal Care and Use Committee (ACUC) at University of California, Berkeley. The LRP5 knockout mice were kindly provided by Dr. Lawrence Chan [14], and were mated with the Sca1-GFP transgenic mice [12] to generate compound mutant mice. The Sca1-GFP was generated as a mouse line where GFP was expressed in all hematopoietic cells [11]; recent studies demonstrated specific GFP expression in the vascular endothelium of skeletal muscle and retina [12], [13].The following primers were used for genotyping the Sca1-GFP transgene: forward-CTGGTCGAGCTGGACGGCGACG and reverse-CACGAACTCCAGCAGGACCATG.

### LacZ Staining and Immunostaining

For LacZ staining, enucleated eyes were fixed with 4% paraformaldehyde, then incubated in PBS containing 0.02% NP-40 and 2 mM MgCl_2_ for 20 minutes, and followed by staining with a LacZ staining solution (2 mM MgCl_2_, 5 mM Potassium Ferricyanide, 5 mM Potassium Ferrocyanide and 1 mg/ml Xgal in PBS) at 37°C for overnight. LacZ stained eyes were post-fixed with 4% paraformaldehyde. The eyes were washed with PBS; retinas were dissected out for flat mount. For frozen sections, LacZ stained eyes were cryoprotected with 30% sucrose/PBS at 4°C before being embedded in Tissue-Tek O.C.T. compound. Nuclei were counterstained with Nuclear Fast Red (Vector Laboratories, Burlingame, CA). Images of LacZ stained retinal whole mount or sections were acquired via a Zeiss Axiovert 200 light microscope with a digital camera.

Immunostaining was performed on ∼10 µm thick frozen sections. A standard protocol was used to examine the cellular localization of different antigens [10]. The following antibodies were used: bipolar cell-specific anti-PKCα (BIOMOL Research Laboratories, Inc., PA), amacrine cell-specific anti-calretinin (Chemicon International, Temecula, CA), horizontal cell-specific anti-calbindin (Chemicon International, Temecula, CA), anti-CRALBP (a generous gift from Dr. John Saari of University of Washington) and anti-glutamine synthetase (GS, BD Biosciences, San Jose, CA) for Müller cells, anti-Tie2 (Calbiochem, La Jolla, CA), and anti-GFAP (DakoCytomation, Denmark).

### Electroretinography

A previously described procedure [24] was used to perform the ERG examination with some minor differences. ERG was recorded by 7-step stimulation with increasing light intensity (0, 0.00032, 0.01, 0.316, 1.0, 3.16 and 31.62 cd.s/m^2^) and prolonged interval between stimulation (30, 30, 30, 60, 60, 120, and 120 seconds). The ERG was recorded by Espion visual testing system (Diagnosys). Each step was repeated 3 times. The average of amplitude values of step 3 (0.01 cd.s/m^2^), step 5 (1.0 cd.s/m^2^) and step 6 (3.16 cd.s/m^2^) from mice of the same genotype were shown in this paper.

### Primary Müller Cell Culture

Müller cells were isolated according to previously described methods [25], [26]. Briefly, eyes from postnatal 5–8 day mice were kept overnight in DMEM medium at 4°C in the dark, followed by one-hour incubation in the dissociation buffer containing 0.1% trypsin (Invitrogen Life Technologies), 70 U/ml collagenase (Type 2, Worthington Biochemical Corporation) in DMEM at 37°C. Retinas were carefully dissected out, chopped into smaller aggregates and cultured in DMEM/10% FCS with penicillin/streptomycin at 5% CO_2_ and 37°C. After 5 days in culture, retinal aggregates and debris were removed by forcibly pipetting medium onto the dish.

### RNA isolation and PCR analysis

Two retinas from each mouse of various genotypes were dissected out, total RNA was isolated using the Trizol® Reagent (Invitrogen Life Technologies), and cDNA was synthesized with the Superscript™ First-Strand Synthesis System for RT-PCR kit (Invitrogen) from equal amount of total RNA for each mouse. PCR was performed on the same amount of cDNA in 20 µl of volume. PCR conditions for all primers sets were: denaturation at 94°C for 2 minutes, 30 cycles of denaturation at 94°C for 30 seconds and annealing and elongation at 68°C for 1 minute, and a final 10 minutes elongation at 72°C. Equal volume of PCR products (3 µl for G3PDH, and 10 µl for all other genes) was loaded for electrophoresis analysis, and amplified DNA fragments were visualized by ethidium bromide in agarose gels. Primers used for PCR amplification were described below. A pair of primers forward-CTTCCACACACCATGCGAGG and reverse-GTCAGACAGGTCATGTACTC amplified a ∼550 bp fragment of LRP5; primer pair forward- CTGGATTATTGTCCCCGGAT and reverse-GTGTGTATTCCAGTCAGTCC generated a ∼460 bp fragment of LRP6; primers forward-CTGCTGCAGTTCCTCCTGCTC and reverse-GGCAAGGGAACCTCTTCATC amplified a ∼600 bp fragment of FZD4; a fragment of β-catenin (∼400 bp) was amplified by the primers forward–ATGGCTACTCAAGCTGACCTG and reverse-GACAACTGCATGTTTCAACATC; the ∼440 bp Slc38a5 fragment was amplified by the primer pair forward-AATGGCCATTTCATGCGCTG and reverse-CAACGTTGTGCAGACAGATG; and a pair of G3PDH (glyceraldehydes-3-phosphate dehydrogenase) primers were used to amply a ∼1000 bp fragment as a house keeping gene control: forward-TGAAGGTCGGTGTGAACGGATTTGGC and reverse-CATGTAGGCCATGAGGTCCACCAC.
